# Development of a peer and near-peer mentoring program to support early career research faculty: The ASPIRE! program

**DOI:** 10.1017/cts.2025.10053

**Published:** 2025-05-30

**Authors:** Stephanie Lovinsky-Desir, Lauren S. Chernick, Brett Anderson, Teresa Lee, Marisa N. Spann, Jennifer Woo Baidal, Gissette Reyes-Soffer

**Affiliations:** 1 Division of Pediatric Pulmonology, Department of Pediatrics, Columbia University Medical Center (CUIMC), New York, NY, USA; 2 Department of Environmental Health Sciences, Mailman School of Public Health, Columbia University Irving Medical Center (CUIMC), New York, NY, USA; 3 Division of Pediatric Emergency Medicine, Department of Emergency Medicine, Columbia University Irving Medical Center (CUIMC), New York, NY, USA; 4 Department of Population and Family Health, Mailman School of Public Health, Columbia University Irving Medical Center (CUIMC), New York, NY, USA; 5 Center for Child Health Services Research, Mindich Child Health and Development Institute, Icahn School of Medicine at Mount Sinai, New York, NY, USA; 6 Department of Population Health Science and Policy, Icahn School of Medicine at Mount Sinai, New York, NY, USA; 7 Department of Pediatrics, Icahn School of Medicine at Mount Sinai, New York, NY, USA; 8 Division of Pediatric Cardiology, Department of Pediatrics, Columbia University Irving Medical Center (CUIMC), New York, NY, USA; 9 Department of Psychiatry, Columbia University Irving Medical Center, New York, NY, USA; 10 Division of Pediatric Gastroenterology, Hepatology, and Nutrition, Department of Pediatrics, Stanford University School of Medicine, Stanford, CA, USA; 11 Division of Preventive Medicine and Nutrition, Department of Medicine, Columbia University Irving Medical Center (CUIMC), New York, NY, USA

**Keywords:** Career development, early stage investigator, institutional support, peer mentoring, women in science

## Abstract

ASPIRE! *(Accountability and Safe-space to Promote, Inspire, Recharge, and Empower)* is a peer mentoring group and peer/near-peer mentoring program established in 2016 by a group of seven early career clinician and non-clinician, research faculty. All founding members participated in the TRANSFORM KL2 Program at Columbia University Irving Medical Center’s Irving Institute for Clinical and Translational Research. In this short communication, we describe the origins of this peer mentoring group established to support these seven early-career KL2 scholars. We also provide a summary of the development of an institution-wide peer mentoring program, created by the seven members of the initial peer mentoring group. We highlight how being at similar career stages, coming from different institutional departments, and sharing common academic goals in a safe space may have contributed to the success of the peer mentoring group. Our individual successes and experiences demonstrate that peer mentoring can be a powerful tool for enhancing the early-career academic experience.

## Introduction

Retention and advancement of diverse faculty, including women, in higher learning institutions has many challenges as highlighted by a study using data from the Association of American Medical Colleges Faculty Administrative Management Online User System database [1]. The barriers to academic retention have been examined by others and include the lack of transparency and trust that faculty have in their own work, a sense of tokenism, challenges in organizational management, and implicit biases [2]. In academic medicine and within the sciences in general, top-down, apprenticeship-like mentoring models have long been used to support faculty learning and development [3–5]. Yet, peer relationships influence the formation of individual or group identity and a sense of belonging [6], leading to recent use in modern mentoring practices [7,8]. Peer mentoring within academic institutions has garnered more recent attention as one potential approach to support faculty growth, academic productivity, and job-life satisfaction [7,8].

Peer mentoring refers to a bi-directional mentoring experience between individuals at a similar career stage who provide support and guidance to one another; while near-peer mentoring refers to a similar mentoring relationship with a peer who may be at a more advanced career stage. Peer [9] and near-peer [10] mentoring can be complementary to traditional types of mentoring and lead to productive and rewarding experiences in academia [11]. Research shows that individuals who participate in peer mentoring: (1) build an enhanced, inclusive, and appreciative culture; (2) have clear career goals, values, strengths, and priorities; (3) have enhanced enthusiasm for collaboration; and (4) develop critical thinking skills [12].

In this special communication, we describe the genesis of one peer mentoring group and the development and refinement of a subsequent peer and near-peer mentoring program for clinician and non-clinician early career investigators at Columbia University Irving Medical Center (CUIMC). We describe the group’s organic roots that began with the National Institutes of Health / National Center for Advancing Translational Sciences (NIH/NCATS) funded KL2 scholars’ program. One goal of the KL2 scholars program is to increase the pool of qualified researchers trained to address challenges in clinical care by providing structured mentoring and training to conduct translational team research. In response to the observed gaps in retention and advancement of academic faculty documented in prior literature [1,13], we developed the peer mentoring group to support one another during the KL2 scholars’ program and throughout our early academic careers. The objectives of the current communication are to: (1) describe the origins of the peer mentoring group that was created to provide support to these seven early career KL2 scholars and (2) provide a descriptive summary of the development of an institution-wide peer mentoring program created by the seven members of the initial peer mentoring group in response to the lessons learned from our academic journey together.

## Origins of the ASPIRE! group

In April 2016, the TRANSFORM KL2 Program at Columbia University Irving Medical Center’s Irving Institute for Clinical and Translational Research, (Clinical Translational Science Award (CTSA) UL1TR000040 and UL1TR001873) included 10 early career faculty scholars (8 women, 2 men; 7 first year KL2 awardees, 2 second year and 1 third year). As part of the training curriculum, scholars attended the annual Association for Clinical and Translational Sciences (ACTS) conference. Stimulated by the sense of belonging felt in this environment, the Irving Institute director and founding members of the ASPIRE! group, at the time KL2 scholars, discussed their shared experiences and challenges in (1) designing research studies, (2) writing grants, (3) writing manuscripts, (4) fulfilling clinical responsibilities, and (5) searching for work/life balance. With a few exceptions, there was consensus that individual productivity, particularly for those in clinical divisions, was largely influenced by (a) the ability to set achievable goals and deadlines, and (b) the need for support with accountability. We described a desire for a safe space to assess unfiltered ideas and concerns. We felt supported by the KL2 program leadership but still felt isolated. This was scary, exhausting, and disempowering.

Following the conference, we—a group of KL2 scholars—recognized that we wanted to thrive in an environment of comradery rather than individualism. We began to convene every Tuesday over lunch to share our progress and hold each other accountable for our writing goals each week. All ten scholars were included, and seven engaged in the weekly writing accountability meetings. One of the seven participants left the institution within the first year and a new KL2 scholar was invited to join the group. Thus, seven early career faculty, each identifying as women, continued to engage in weekly meetings over 2 years. Among our group, one individual identifies as Hispanic, two as non-Hispanic Black, one as non-Hispanic Asian, one as biracial (Hispanic and Asian), and two as non-Hispanic White. We are affiliated with six different divisions and four academic departments, and collectively bring expertise in bench research, clinical practice, and population health. The initial goals of the ASPIRE! peer mentoring group were to support one another through a challenging phase in our academic careers and to encourage one another to maintain scholarly productivity through publishing manuscripts and securing additional grant funding. The purpose of our initial meetings was to enhance writing accountability, a key metric to academic success and early career development. During our weekly meetings, we shared writing goals and provided accountability, stating our week’s goals aloud and sharing updates on completed tasks and barriers. The tasks were documented and updated weekly in a shared Excel file. The overall success of our program was defined as academic productivity and measured in the number of grants funded and academic promotions.

**Figure 1.**
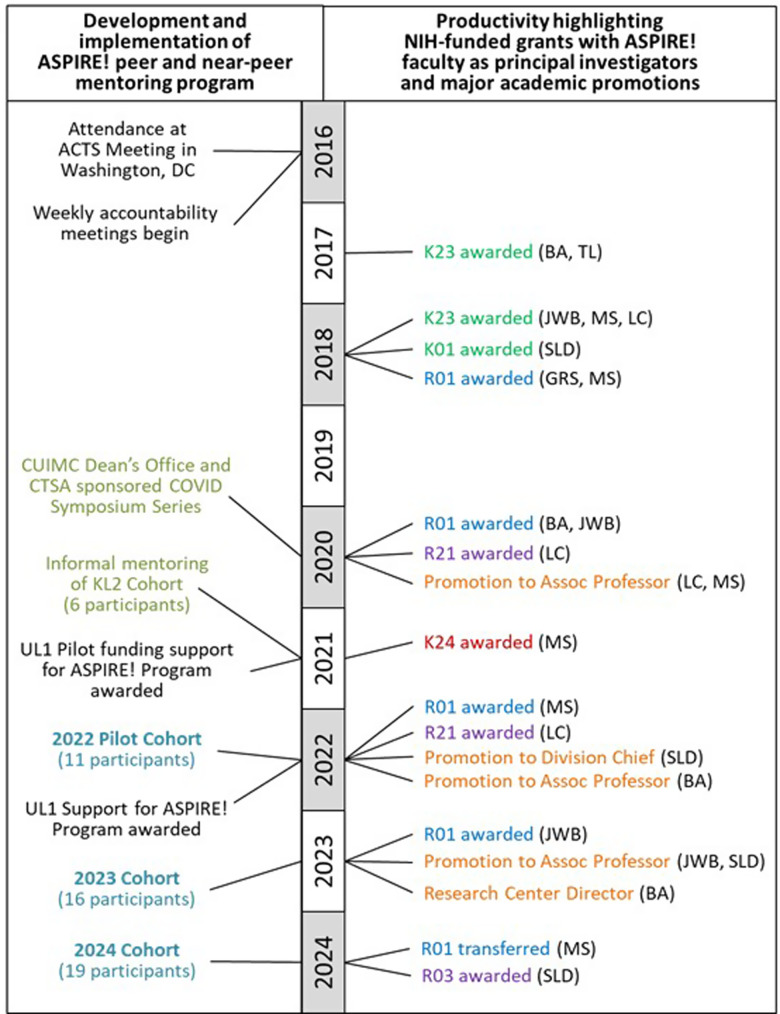
Time-line of development and implementation of ASPIRE! peer and near-peer mentoring program and productivity highlighting NIH-funded grants with ASPIRE! faculty as principal investigators and major academic promotions.

Within the first 2 years of the development of the weekly writing accountability meetings, all seven members received their first external grants (K23/K01 or R01 awards, Figure 1). Concurrently, the weekly meetings became more than a place for accountability; they became a confidential, safe space to share concerns about challenging situations encountered in both work and personal lives and to obtain advice from peers who were managing similar career challenges. We discussed how to collaborate with senior mentors and be a senior mentor (mentoring up and down), how to navigate grant and promotion processes, research hurdles, clinical obligations, and the multi-tasking of life. We shared grant opportunities and our previously accepted applications. We recommended each other for speaking engagements, advised on salary negotiations, and promoted each other on social media. We edited each other’s writing samples, celebrated published manuscripts and successful applications, consoled rejections, and inspired innovative ideas, learning from each other, and enhancing collaborations.

As stated, one of the measures of success of our peer mentoring group was productivity in research grant funding. To quantify the achievements of the members of our peer mentoring group compared to other KL2 scholars we compared success rates of NIH grant funding for the seven ASPIRE! group members to KL2 scholars who were recruited from 2013–2017, overlapping with the period when ASPIRE! members were recruited to the KL2 (2014–2016). The success rate for securing additional NIH funding (number of K, R or U-level grants divided by number of scholars) was 1.8 for ASPIRE! group members compared to 1.3 for the twenty-three other KL2 scholars recruited between 2013–2017. These findings suggest that the academic productivity of our group outpaced other KL2 cohorts at our institution.

**Figure 2.**
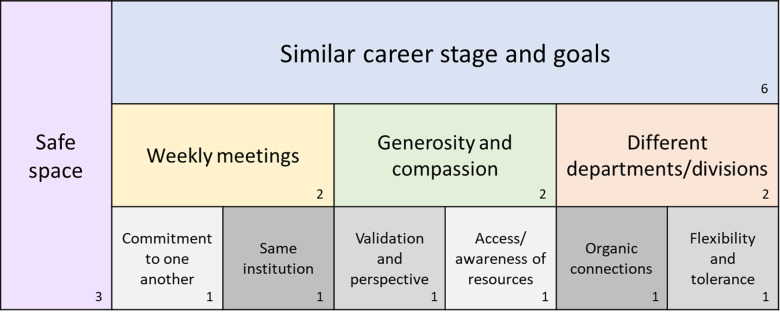
Informal, anonymous survey results from the seven ASPIRE! group members in response to the question: “What were the 3 most important aspects that contributed to the success of our peer mentoring group? ” The size of each box is meant to represent the proportion of that response relative to the total number of responses.

The structure and components of our peer mentoring group align with the National Center for Advancing Translational Sciences (NCATS) 7 Translational Science Principles as outlined in Table 1 [14]. An informal, anonymous survey was conducted among the seven original ASPIRE! group members to identify individuals’ impressions of the factors that contributed to the success of our peer mentoring group; with success defined as academic productivity in grant submissions and promotions as outlined in Figure 1. Group members were asked to provide their opinion on the top three most important aspects (i.e., the “secret sauce”) that contributed to the success of the peer mentoring group. Six of the seven members believed that being at a similar career stage with similar career goals were among the key components of success (Figure 2). Additionally, group members felt that it was important to have a safe space that allowed for trust, generosity, compassion, commitment to one another, sharing of resources, validation, and perspective. Group members also felt that weekly meetings provided flexibility for people to join as schedules permitted and being members of different departments and divisions within the same institution contributed to the success of the group (Figure 2). A future, in-depth analysis of the key drivers of success for our ASPIRE! group is forthcoming.

**Table 1.** The ASPIRE! peer mentoring group’s approaches to addressing the National Center for Advancing Translational Sciences 7 translational science principles

Translational science principle	ASPIRE!’s approach to meeting the principle
*Prioritize initiatives that address unmet needs*	Provides a safe space for mentorship for early career faculty, including opportunities to address personal challenges
*Produce generalizable solutions for common and persistent challenges*	Supports retention and successful promotion of diverse early career faculty in academic settings
*Emphasize creativity and innovation*	Multi-faceted opportunities for engagement through peer mentorship include sharing weekly writing goals, exchanging resources, and developing new programing together
*Leverage cross-disciplinary team science*	Faculty represented from multiple departments and divisions providing diverse perspectives
*Enhance the efficiency and speed of translational research*	Weekly sessions and personal connections provide opportunity for frequent feedback and troubleshooting
*Utilize boundary-crossing partnerships*	Collaboration and partnership with institutional leaders from various departments to develop novel, institution-wide, peer mentoring programing
*Use bold and rigorous research approaches*	Creating new institutional programs to support early career faculty and developing peer mentoring groups across the institution using knowledge gained from our group

## Development of ASPIRE! programs within our institution: Sharing our success with others

**Figure 3.**
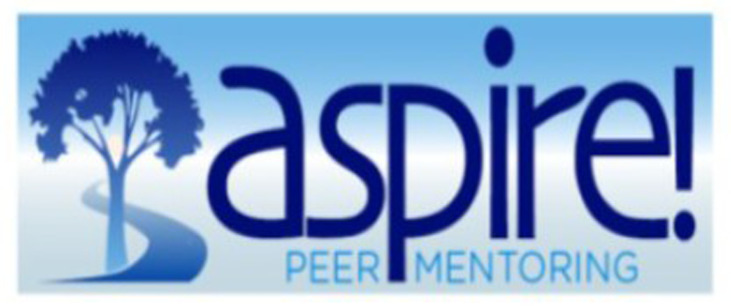
ASPIRE! group logo.

After two years of holding regular weekly meetings, we observed consistent and significant academic growth among our members, along with a noticeable sense of personal fulfillment. A couple of the founding members had been involved in other peer mentoring groups, but this was something different. In the Spring of 2019, we convened our first retreat to develop a consensus statement on what our group meant to us, why we believed it was so impactful, and how we might consider sharing it with others. To encapsulate these themes, we branded ourselves with the name ASPIRE! (**A**ccountability and **S**afe space to **P**romote, **I**nspire, **R**echarge and **E**mpower!). Our mission statement articulated future goals to strengthen the pipeline of interdisciplinary faculty researchers across ranks, tracks, and departments, and create a spirit of collaboration and networking amongst early, mid-level, and senior faculty members through peer mentoring teams, with a focus on the advancement of women and under-represented groups. Our focus on equity stemmed from the demographic make-up of the individuals who were in the group and the well-established literature on disparities in academic advancement for women and under-represented groups in the sciences [13,15–18]. Following the retreat, we developed a logo, as seen in Figure 3, to represent the wide-reaching, inter-woven, peer roots supporting the journey in front of us and the growing branches (7 branches) representing each of the founding ASPIRE! members.

We also recognized the need to create key partnerships within the institution to support our vision of expanding our work in peer mentoring. ASPIRE! began conversations with the leaders of Columbia’s CTSA and the Office of Academic Affairs/CUIMC Dean’s office, to discuss opportunities to advance and expand mentoring within our institution, and our “secret sauce,” for success beyond the KL2 program. Importantly, institutional leaders sought to understand more about the success of our writing group whose members had continued to achieve additional successes (independent R01s, internal CUIMC Irving Scholar Awards, Robert Wood Johnson - Amos Medical Faculty Development Awards and NIH Loan Repayment awards - Figure 1), all within three years of completing the KL2 program. In addition to research funding success, the group’s academic productivity and national presence included many peer-reviewed publications, invitations to present at national academic meetings, and membership on advisory boards for journals and foundations.

**Figure 4.**
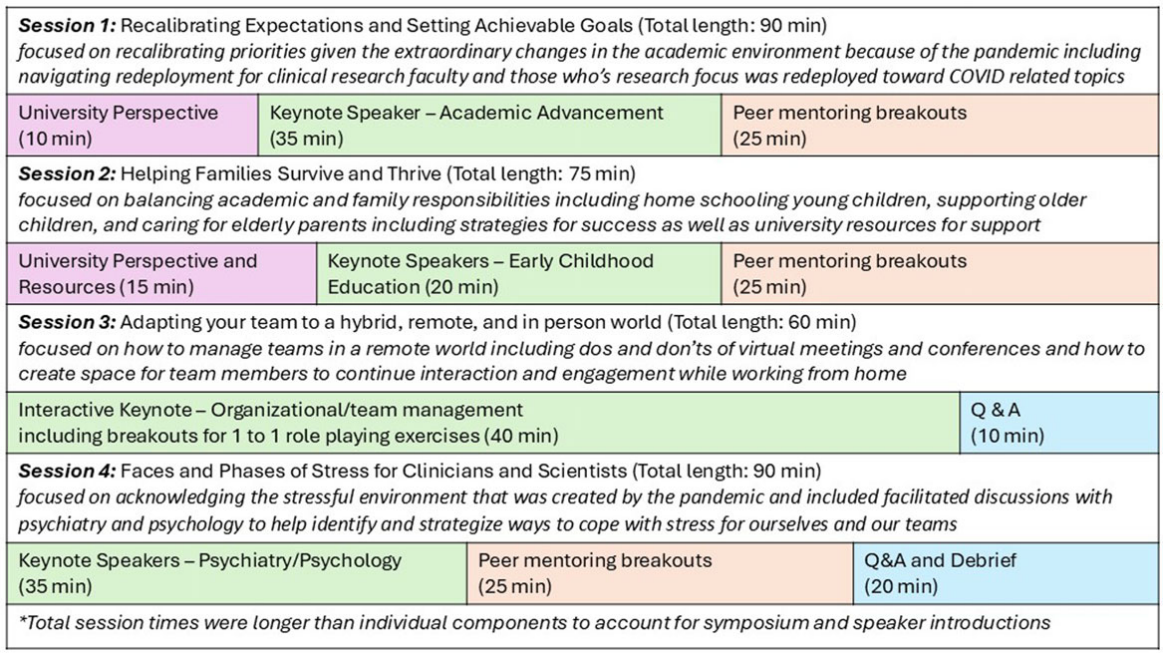
Outline of major components of the 2020 ASPIRE! virtual symposia series designed to provide group peer mentoring to early and mid-career faculty during the COVID-19 pandemic.

In the Summer of 2020, in the wake of the clinical disaster and research shutdown that COVID-19 inflicted on New York City, we worked with the leaders of Columbia’s CTSA and the Office of Academic Affairs to develop and execute a virtual, peer mentoring symposium series to support early and mid-career academic faculty investigators, a first opportunity to test our ability to support other faculty. We ran four workshops that addressed: (1) Calibrating Expectations, (2) Helping Families Thrive, (3) Managing Remote Teams, and (4) Faces and Phases of Stress (Figure 4). Each session included invited speakers who were subject-matter experts (e.g., psychiatry/psychology, early childhood education, organization/team management, academic advancement) to deliver key messages to attendees. Forty to seventy faculty members attended each session from across the medical center campus. In each session, we provided brief introductions, administrators from the Office of Academic Affairs updated participants on their newest policies and resources, invited speakers discussed actionable solutions, and then ASPIRE! members facilitated small-group, breakout sessions.

The success of the workshop was reflected in attendee surveys, which revealed that (a) the peer-to-peer breakout sessions enhanced learning opportunities, and (b) peer support helped address feelings of isolation, challenges with self-accountability, and the need for safe spaces and community support. This feedback reaffirmed that the feelings that led us to start ASPIRE! were not unique to the founding members and suggested that if we developed additional programing, we could help other faculty find crucial support. Additional feedback included (1) the importance of including experts in each session that focused on actionable solutions and stimulated the most productive conversations during breakouts, (2) including groups of peers from different disciplines and career stages provided a range of actionable recommendations for institutional leaders, (3) the greatest learning was obtained from peer-to-peer breakout group sessions. Data on this symposium were presented at the 2021 ACTS Annual Meeting [19].

In early 2020, inspired by our ASPIRE! programing, a group of junior current KL2 scholars formed a spin-off writing accountability and peer mentoring group called ASPIRE!2. The original ASPIRE! group members served as near-peers, joined the new cohort’s meetings monthly, met with faculty individually as needed, and shared advice and opportunities over email. In 2021, the CUIMC CTSA leadership allocated funding for the ASPIRE! founding group members to conduct a pilot series of peer mentoring workshops for the newest cohort of KL2 scholars and ASPIRE!2. We created three 1-hour workshops, paired with a 30-minute peer mentoring session, that covered areas including elevator pitches, building mentoring relationships, motivational factors within academic careers and developing a roadmap to success. Participants completed electronic feedback surveys after each of the three sessions. The results of the surveys highlighted that: (1) the topics were applicable to the needs of early-career research faculty, and (2) there was enthusiasm for the engagement of ASPIRE! founding members in small group sessions as near-peer mentors. In addition, the participants requested opportunities for formalized peer mentoring teams that could continue beyond the pilot programing year.

**Figure 5.**
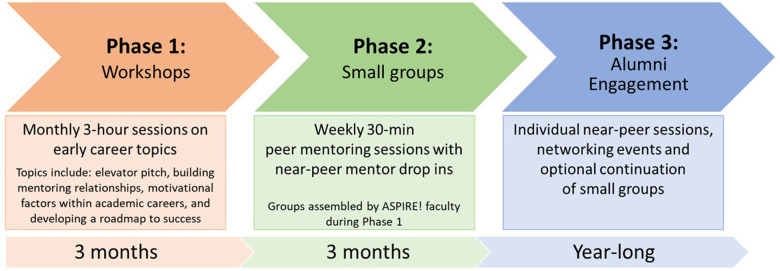
Timeline of institution-wide ASPIRE! program peer and near-peer mentoring activities.

After successful completion of our pilot program in 2021–22, the ASPIRE! founding group members received funding under the TRANSFORM CTSA grant to expand the scope of the peer mentoring program throughout the CUIMC campus in the 2022–23 academic year. Feedback from the pilot programs informed the structure of workshops and peer mentoring sessions as well as the content for the new workshops. We incorporated feedback regarding the importance of creating peer mentoring groups that could extend beyond the workshops and incorporating near-peer mentoring. The content covered in the current workshops aligns with the needs and feedback that were elicited during the pilot phases. The purpose of the newest iteration of the program is to create formalized opportunities for peer and near-peer mentoring for early career clinician and non-clinician research faculty across the medical center campus. The implemented program includes three phases (Figure 5). Phase 1 includes three 3-hour workshops over a 3-month period. Workshops focus on essential topics: (a) refinement of elevator pitches (i.e. defining your academic identity) as a means of facilitating peer bonding, (b) identifying resources for growing research teams, (c) strategic career planning and writing, and (d) how to be “deliriously happy” at work [20]. Phase 2 includes weekly 30-minute peer mentoring sessions with small groups of 5-7 participants over a 3-month period. Peer mentoring sessions offer a chance to share writing goals, challenges, and successes. They are designed to reflect the spirit of what originally brought the seven founding members of ASPIRE! together. ASPIRE! founding members also periodically participate in the small group peer mentoring sessions to provide near-peer mentoring and facilitate forthright conversations. In Phase 3, we invite participants to continue to meet in their small peer mentoring groups as well as to engage with other program alumni and ASPIRE! founding members (Figure 5). To date, three cohorts have completed the full ASPIRE! Peer Mentoring Program; and overall, the ASPIRE! program has engaged over fifty early career investigators across nine departments at CUIMC School of Medicine and Public Health. Program outcomes, including the results of a formalized early career needs assessment and program satisfaction, are being measured and will be reported in future publications.

Importantly, the ASPIRE! group’s founding members continue to meet weekly to provide mid-career peer mentoring and support. During these weekly sessions, there are 30 minutes of “life catch up” and 30 minutes of writing accountability.

## Discussion

In this special communication, we report the origins of a peer mentoring group and the subsequent development of a peer/near-peer mentoring program that aims to provide early career investigators a safe space, accountability, and sense of belonging. We highlight the founding members’ journey and successes that came from the ASPIRE! group and the implementation of programing across a large academic institution.

To our knowledge, there is no data on the prevalence of peer mentoring programs at academic institutions. The few manuscripts that have documented successful peer mentoring programs are notably different from the ASPIRE! group because: (1) the interval between meetings was longer, ranging from 1–4 months, (2) sessions were facilitated by a senior faculty member, and (3) groups included faculty within the same departments or research areas of interest [3,7,8,12,21,22]. Our weekly meetings, organic connections without senior faculty involvement in sessions, and multi-disciplinary and diverse department representation were key to the success of our peer mentoring group and set the stage for our future institutional programs. We encountered several challenges in the early stages of our peer mentoring group including navigating scheduling conflicts for the weekly meetings, finding appropriate spaces to meet regularly, navigating the challenges of mentorship and challenges experienced during the COVID-19 pandemic. We have outlined in Table 2 some of the strategies we adopted to overcome these challenges.

**Table 2.** Barriers and challenges experienced during the formation and early years of the initial ASPIRE! peer mentoring group

Barrier/challenge	Solutions
Meeting schedule conflicts	Maintained weekly meeting structure, members could join as they were able Group was large enough (7 members) so there were always some who could attend Recorded weekly writing goals in a shared document so those not in attendance could still provide updates
Identifying locations for meetings	Met outdoors when weather permitted Requested support from CTSA for a meeting room
Mentors leaving the institution/ challenging mentoring relationships	Facilitated networking with new potential collaborators and mentors Shared laboratory space, personnel and resources with one another Provided support
Changes during the COVID-19 pandemic that affected professional and personal lives	Created a campus-wide symposium series to provide resources and guidance that benefited our members and our peers across the institution

Through the process of forming our own group, we learned valuable lessons that were key to informing the development of subsequent institution-wide programs. The top factors that contributed to our success, according to the founding members, were similar career stage and goals, safe space, weekly meetings, and multi-discipline/multi-department membership; all factors that can be replicated to support similar programs at other centers and institutions. The multi-disciplinary, multi-department nature of the ASPIRE! group provided essential lessons and diverse insight that were key in the development of the peer/near-peer group mentoring strategies. The focus on short-term writing and long-term career goals during weekly meetings allowed our peer mentoring teams to hold one another accountable to those goals, without feeling pressure from leaders and peers within our departments and divisions. We came to appreciate the importance of safe spaces where small groups of faculty members can have a dialog with those that may be outside their division and/or discipline. These groups allow free conversations about challenges and best practices that are not always transparent. Being from unique spaces within a large academic institution provided a balance of closeness and distance that was likely foundational to the founding ASPIRE! members’ academic accomplishments. Additionally, we found that attending regular meetings with colleagues outside of our discipline but at a similar career stage provided inspiration for new project ideas, funding opportunities, and problem-solving approaches. We also found that groups of 5–7 peers (as opposed to smaller or larger groups) allowed for continuity and quorum even as busy schedules did not allow everyone to join each meeting.

The above-mentioned lessons learned and the structure of our peer mentoring group align with the 7 core principles of translational science that have been developed to support the process of translating scientific discoveries to improve patient care and public health [14]. As early-career academic research faculty, the founding ASPIRE! members realized that to move forward meaningful science and maintain academic productivity, we needed to create support structures within our institution that would enable us to be successful. The recognition of our own academic successes led us to the process of developing an institution-wide peer and near-peer mentoring program for early career faculty. Additional data on the outcomes of the larger program is forthcoming.

We acknowledge several limitations of the data presented in this short communication. First, we describe the genesis of a peer mentoring group that was created organically. We recognize that the strong connections that were created over time may be difficult to replicate in other groups and other settings. However, data from our pilot phases and the ongoing institution-wide program suggest that many of the benefits that we experience in the original peer mentoring group can be replicated. Additionally, as the intention of the short communication was to provide a narrative description of the group and program development, we provide limited outcomes and data here. Yet we do include both quantitative data that demonstrates the successes of the members of the group and qualitative data that describes the lessons learned. We believe this information may be useful to others who may be interested in developing and implementing a similar program. Lastly, we are unable to provide a detailed analysis of alternative approaches to our peer mentoring strategy as this is outside the scope of this short communication.

As the ASPIRE! program evolves, we continue to promote and amplify each other by sharing resources, nominating each other for talks, and providing a sounding board for innovative ideas and proposals. In our ongoing work, we continue to evaluate the successes and opportunities for improvement with the institution-wide program through formalized feedback with cohort participants, including considerations to expand across other institutions.

## Conclusion

This short communication describes the development of a peer/near-peer mentoring effort by seven KL2 scholars. The highlights include the individual successes and experiences of the scholars and demonstrate that peer mentoring can be a powerful tool for enhancing the early academic experience.
